# Why is transition between child and adult services a dangerous time for young people with chronic kidney disease? A mixed-method systematic review

**DOI:** 10.1371/journal.pone.0201098

**Published:** 2018-08-02

**Authors:** David J. Dallimore, Barbara Neukirchinger, Jane Noyes

**Affiliations:** School of Social Sciences, Bangor University, Wales, United Kingdom; Weill Cornell Medical College in Qatar, QATAR

## Abstract

Young people age 14–25 years with chronic kidney disease have been identified as generally having poor health outcomes and are a high-risk group for kidney transplant loss due in part to poor self-management. This raises a key question as to what happens during transition from child to adult services? This paper presents a mixed-method systematic review of health and social care evidence concerning young people with chronic kidney disease transitioning from child to adult health and social care services. Quantitative and qualitative evidence were synthesised in streams followed by an overarching synthesis. Literature searches (2000 to March 2017) were conducted using Pubmed, BioMed Central and Cochrane Library, grey literature sources ZETOC, .gov.uk, third sector organisations, NHS Evidence, SCIE, TRIP, Opengrey. Snowball searching was conducted in the databases Ovid, CINAHL, ISI Web of Science, Scopus and Google Scholar. Of 3,125 records screened, 60 texts were included. We found that while strategies to support transition contained consistent messages, they supported the principle of a health-dominated pathway. Well-being is mainly defined and measured in clinical terms and the transition process is often presented as a linear, one-dimensional conduit. Individual characteristics, along with social, familial and societal relationships are rarely considered. Evidence from young people and their families highlights transition as a zone of conflict between independence and dependency with young people feeling powerless on one hand and overwhelmed on the other. We found few novel interventions and fewer that had been evaluated. Studies were rarely conducted by allied health and social care professionals (e.g. renal social workers and psychologists) as part of multi-disciplinary renal teams. We conclude that there is a lack of good evidence to inform providers of health and social care services about how best to meet the needs of this small but vulnerable cohort.

## Introduction

Medical advances have led to increased survival rates for children and adolescents age 0–19 years with chronic kidney disease (CKD) [1]. The outcome of kidney transplantation has generally been improving since the 1990’s [2,3]. Yet, adolescents (age 14 to 19 years) and young adults up to around age 25 years (collectively referred to as young people in this paper) experience higher levels of kidney transplant loss (also called graft loss) than either younger children or older adults [4–6] and are generally identified as a particularly high-risk group for poor outcomes. While it has been suggested that maturation of the immune system and increased growth during adolescence (14–19 years) may contribute to rejection of the transplanted kidney [7], non-adherence or discontinuation of immunosuppressive medications has been identified as the major contributor to graft loss among older adolescents and young adults [4,8]. Young people age 14–25 years with CKD, who have not yet received a kidney transplant, are also known to have poorer outcomes than younger children or older adults in the same situation. This raises a key question as to what happens during transition from child to adult services from age 14 to 25 years? An initial scoping search identified no relevant systematic reviews that took a holistic health and social care perspective to the transition of young people with CKD. Although there are systematic reviews of transition and other long-term conditions, which may provide some useful overarching common insights, young people with CKD have very specific medical needs and experience their condition in distinctive ways. For example, steroids may dramatically alter their appearance and mood, CKD may stunt their growth, lifestyle modifications may be extreme (such as fluid and diet restrictions), medications may have unpleasant side effects and treatments (such as dialysis) can be very time consuming and restrictive, and depending on the modality may involve employed or unpaid carers, and regular travel to hospital. CKD may also impact on reproductive choices and lifestyle ambitions as well as education options, such as choice of school, university or apprenticeships, career choices, travel, relationships and ability or choice to live independently in adulthood. In addition, the configuration of renal services is unusual in that health and key social care services are generally integrated within a single multi-disciplinary team, often located in a tertiary hospital (see for example, Box 1) and young people living in the community with CKD may have to make regular trips to distant centres for ongoing monitoring and treatment. We therefore set out to undertake a systematic review to address the knowledge deficit in this area.

**Box 1: Example of key members of the multi-disciplinary team co-located in a tertiary hospital setting**

Nephrologist (Kidney Doctor)

Renal Surgeon (Kidney Surgeon)

Transplant Surgeon (Carries out kidney transplants)

Advanced Nurse Practitioner–specialising in caring for patients with kidney disease

Renal Nurse Specialist–specialist in kidney-specific aspects of care such as dialysis.

Renal Nurse

Renal Dietitian

Renal Psychologist

Renal Social worker

Renal Pharmacist

Renal Counsellor

Renal technicians–who work in dialysis centres

Vascular Access Team–who do minor surgery on an arm, leg, neck or upper chest to create an access for treatments such as haemodialysis (Surgeon, radiologist and nurse)

Our aim was to investigate the effectiveness of interventions supporting the transition from child to adult services for young people with chronic kidney disease.

1. Our objectives were to:
2. Identify from published studies the specific transition needs and problems experienced by young people with CKD aged 14–25 years.
3. Determine the effectiveness, cost effectiveness and wider impacts of interventions to support transition for young people with CKD.
4. Explore the views and experiences of young people age 14–25 years with CKD and their parents/families and professionals of transition generally and interventions to support transition specifically.

The purpose of this paper is to report the review findings and to highlight the significant gaps and to identify opportunities for subsequent cross disciplinary research.

## Design and methodology

### Theoretical approach

**Fig 1:**
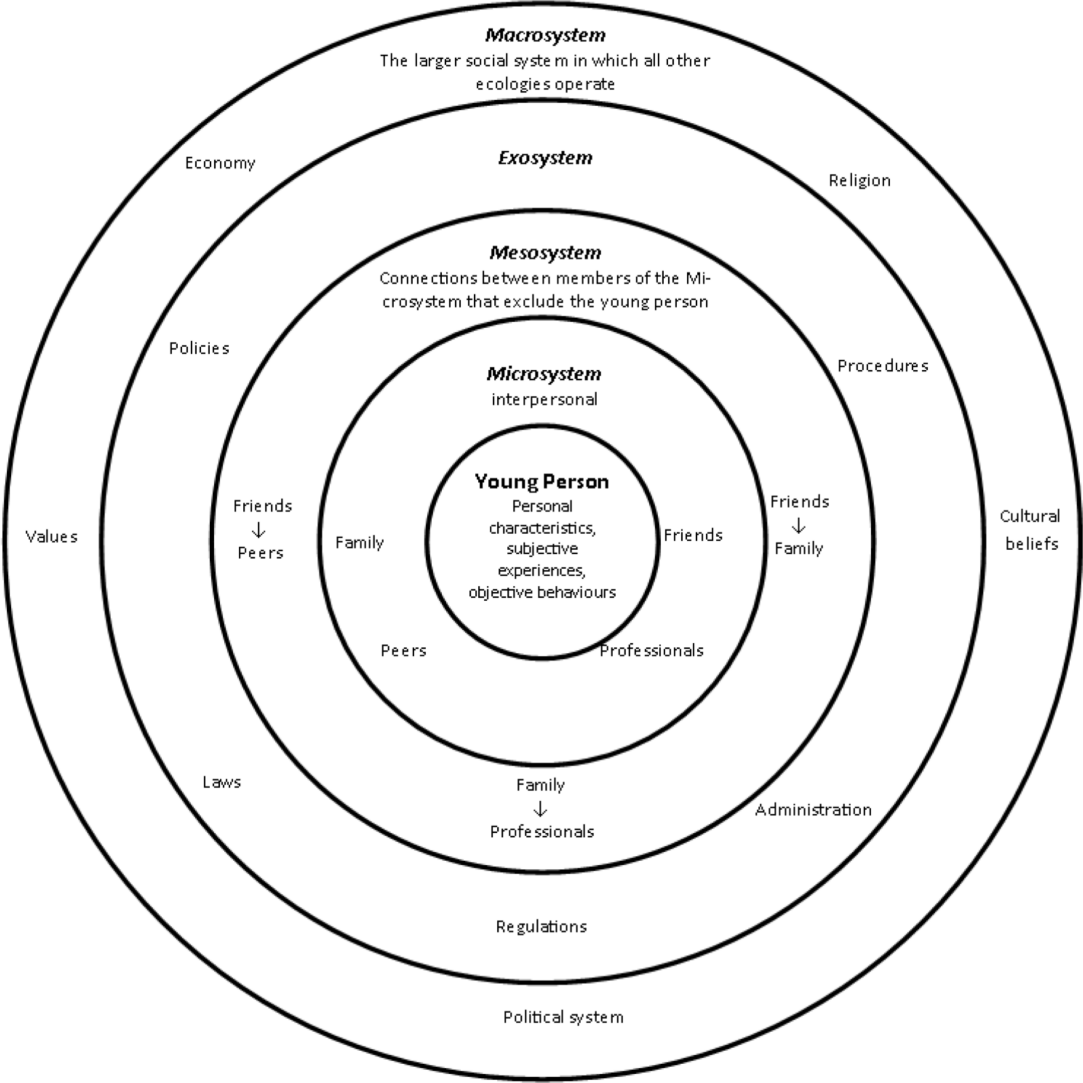
Bronfenbrenner’s [12] Bioecological theory as applied to young people.

Social theory has an important role in systematic reviews to provide the ‘lenses’ through which complex issues can be understood. In this review, two theories, Bronfenbrenner’s Bioecological Model and Meleis’ Transition Theory were used to underpin review processes (such as data extraction) and create an interpretive lens for synthesising and understanding the evidence. Relevant studies [9,10] have drawn upon the bioecological model proposed by Bronfenbrenner [11] to describe a system with multiple environments that surround an individual and therefore require consideration when examining the transition of young people from child to adult services. According to Bronfenbrenner [11], features of environments relevant to an individual's development include both its objective properties and the way a person subjectively experiences these properties. For a young person with CKD and experiencing transition, the model places them at the centre of several distinct environments (see Fig 1) with increasingly complex, reciprocal interactions which can have both direct (e.g. procedures and treatment) and indirect (e.g. policies and laws) influence [12]. We also used Meleis’ [13] Transition Theory as a way of integrating the promotion of health transitions informed by an understanding of the properties of transition (transition conditions) and the promotion of environments within which developmental outcomes are nurtured.

### Review design

**Fig 2:**
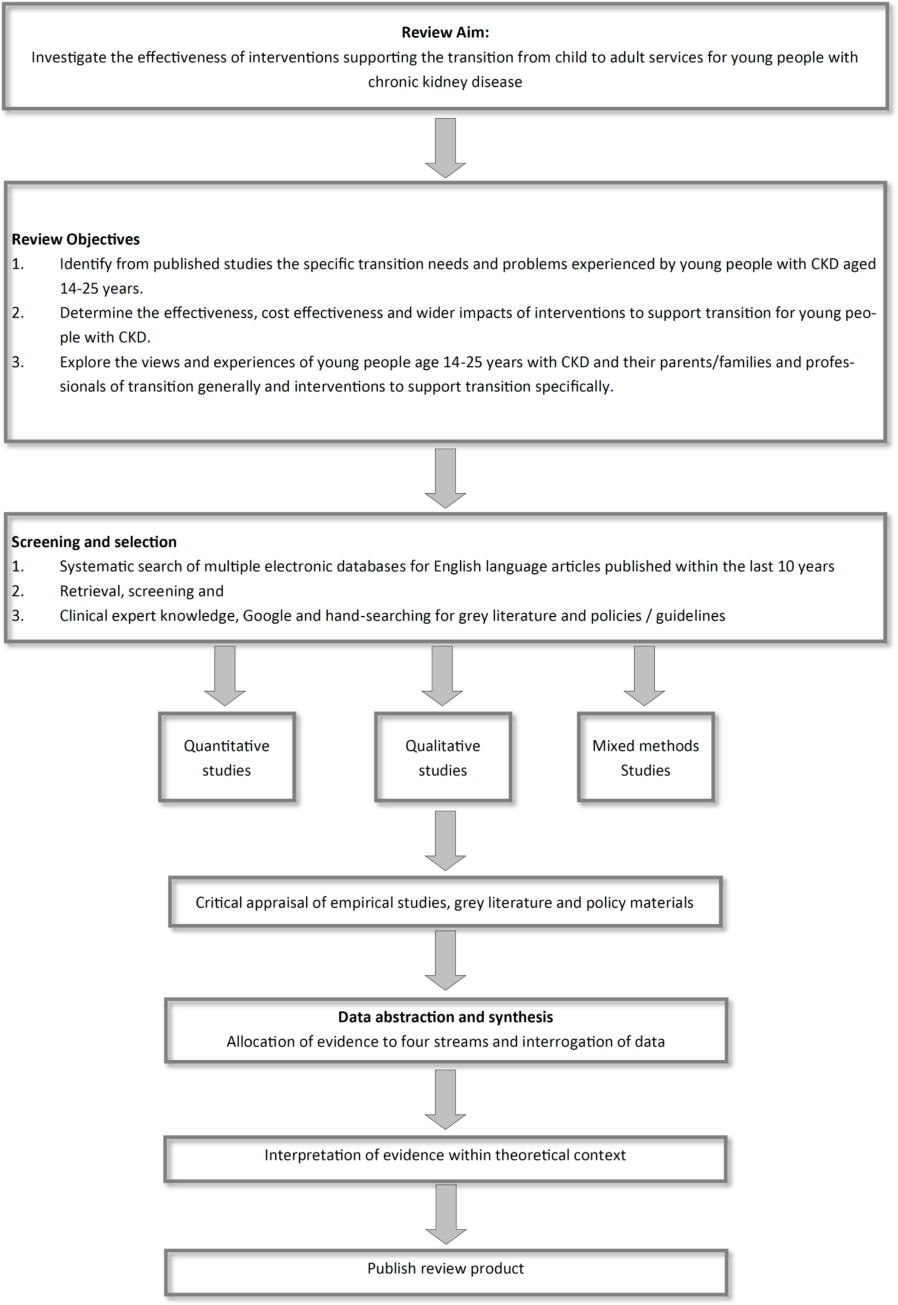
Review design.

We conducted a mixed-method systematic review (Fig 2), adapting principles of the mixed-method synthesis design used by the Evidence for Policy and Practice Information and Coordinating Centre (EPPI, n.d.). Separate streams of evidence were created for each of the three objectives followed by an overarching synthesis.

#### Searching

Searches were designed to identify relevant studies across the three streams of evidence from year 2000 onwards to March 2016, published in English. The inclusion criteria and SPICE search framework (Setting, perspective, phenomena of interest, comparison and evaluation) for each stream is set out in Table 1. As this review was interested in a holistic view of transition issues, studies crossed disciplinary boundaries of health, social care, social science and psychology. Studies of grey literature such as reports from health and third sector organisations and governments were included to limit publication bias and to ensure that all relevant literature was located [14].

**Table 1:** SPICE search strategy.

*Stream of enquiry*	Search terms	Inclusion criteria	Setting	Population / Perspective	Intervention/ Phenomenon of Interest	Comparison	Evaluation
1. The transition needs of young people	(kidney* or renal or nephrol* or *dialysis or dialys*) AND ("Outcome Assessment (Health Care)" OR "Patient Outcome Assessment" OR "Treatment Outcome" OR "Health Services Research" OR "Patient Preference" OR "Evidence-Based Practice" OR "Quality-Adjusted Life Years" OR "Controlled Clinical Trial" [Publication Type] OR "Causality" OR "Epidemiologic Factors" OR "Cohort Studies") OR "Risk" OR "Retrospective Studies" OR "Prognosis" OR "Mortality" OR "Follow-Up Studies" OR "Long Term Adverse Effects") AND transition to adult care ("Qualitative Research" OR "Focus Groups" OR "Hermeneutics" OR "Anthropology, Cultural") OR "Grounded Theory") OR "Peer Review, Research") OR "Health Services Research") OR "Social Validity, Research")) AND ((("Kidney Diseases" OR "Renal Replacement Therapy" OR (kidney[All Fields] OR nephrol*[All Fields] OR renal[All Fields] OR *dialysis[All Fields] OR dialys*[All Fields]) or "Transplantation")	Studies that refer to CKD and young people focused on the concept of transition alongside other possible issues (medical, social, psychological etc.) that initial scoping suggests may be relevant.	The developed world (with comparable health systems to the UK)	Young people aged 14–25 years with CKD	Interventions and strategies aimed at supporting transition for young people with CKD	Comparison of outcomes to determine effectiveness. Different views, experiences and perceptions of young people and key stakeholders.	Controlled intervention studies, before and after studies, intervention studies with no control, validation studies with or without control Qualitative studies
2.Interventions to support transition		Studies that propose, report on or evaluate interventions to support the transition of young people with CKD from child / paediatric to adult health service.	Health, social care, educational and other settings in which young people are supported.	Young people aged 14–25 years with CKD	Interventions to improve transition from child to adult health and social care services	Modified support [or usual care ]	Controlled intervention studies, before and after studies, intervention studies with no control, validation studies with or without control Graft loss Perceived level of social care support Well-being indices Health outcomes
3. The views of children and young people of transition	(youth or young or "emerging adult* OR adolesc* OR teen* or pube*) AND (Transition* OR transfer* AND (kidney OR renal OR nephrol* OR *dialysis OR dialys* OR transplant*) filter for social care	Reports and studies that present the views of young people with CKD; of families of young people with CKD; and of professionals (health and non-health) working with young people with CKD.	The developed world (with comparable health systems to the UK)	Young people aged 14–25 years with CKD	Transition from child to adult health and social care services	None	Evidence of experiences and outcomes related to social, psychological, educational, economic and political life

#### Data sources

A very broad search for the concept of transition AND terms relevant to kidney disease, transplantation, nephrology, or renal replacement therapies was carried out (see Table 1). The databases searched included subscribed databases (e.g. Web of Science, Cinahl etc.) and open access databases (Pubmed, BioMed Central, Cochrane Library). The grey literature was searched through ZETOC, databases that collate conference proceedings (e.g. Cinahl, internet searches of relevant domains such as .gov.uk, third sector organisations, NHS Evidence, SCIE, TRIP, Opengrey). Internet search terms were ‘hedged’ with keywords (e.g. policy, pathway, project etc.) and then some ‘snowball searching’ of key documents was added and judicious ‘seed pearling’ was carried out on a limited number of key documents.

#### Screening

**Fig 3:**
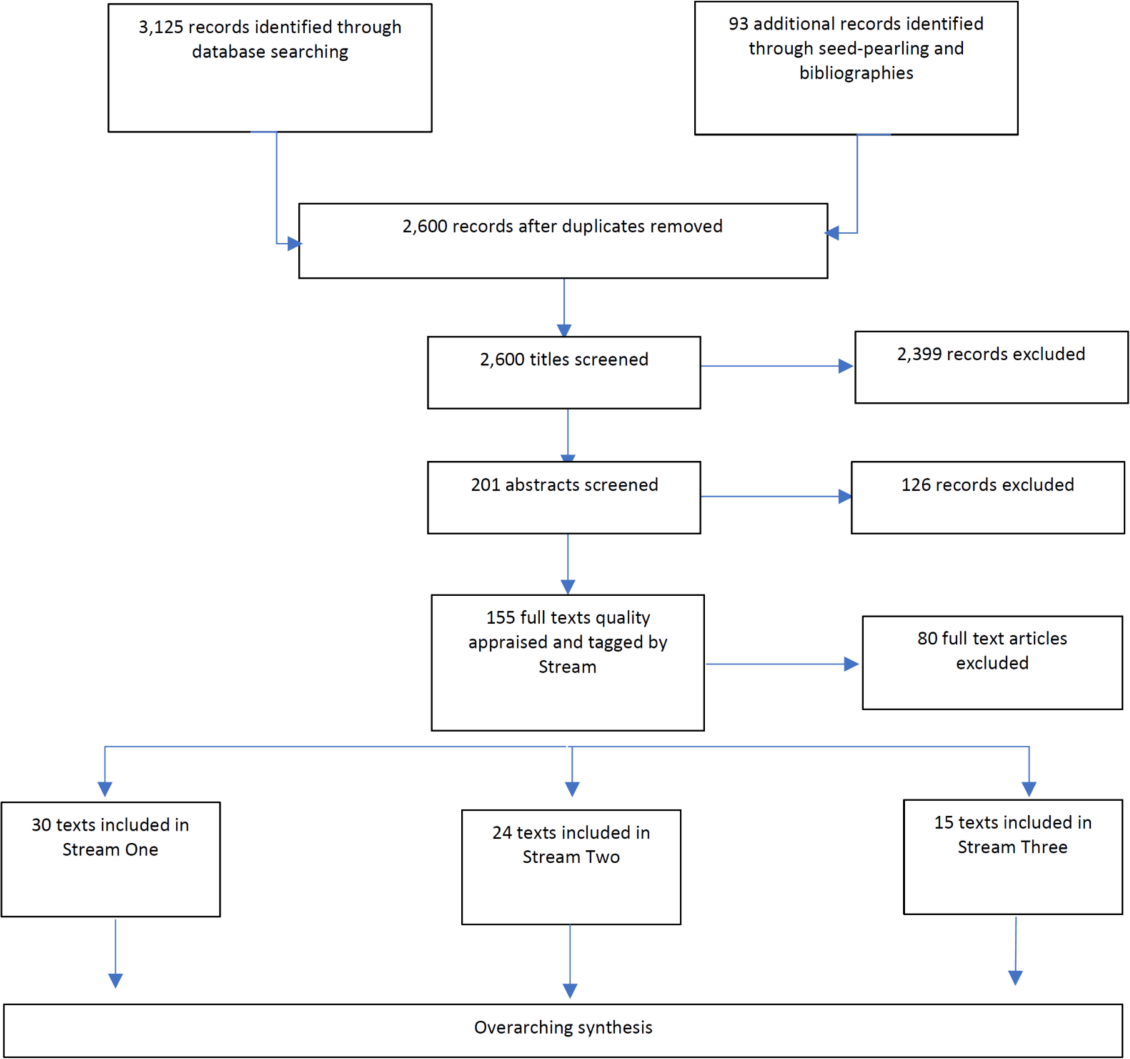
Study selection process flow diagram.

Titles were collated and de-duplicated using Mendeley bibliographic software (https://www.mendeley.com). Titles, and then abstracts were screened against the stream specific inclusion/exclusion criteria by reviewers. The full texts of these articles were obtained, further reviewed and tagged per the potential Stream of Evidence. Remaining studies were evaluated from the full text by two independent reviewers for final inclusion/exclusion within each Stream. At each stage of screening, discrepancies between reviewers were resolved by discussion and any disagreements resolved by discussion with a third team member. Multiple articles from the same study were linked together and in a small number of cases, studies were allocated to more than one Stream of Evidence. The Flow Diagram (Fig 3) details the study selection process.

#### Quality appraisal

Full text documents were appraised using the appropriate tool for the research methodology (Table 2). As with other stages, second readings provided reviewers with opportunities to scrutinise papers against the inclusion criteria for each research question. Included studies were separated into the streams of evidence and critically appraised by two independent reviewers for methodological limitations. A summary of all included studies is provided (S1 Table). No studies were excluded on the basis of methodological strengths and limitations. Any methodological limitations were however taken into account when developing the findings and are acknowledged in the discussion.

**Table 2:** Quality appraisal tools used for each type of evidence.

Type of Study	Quality Appraisal Tool
Randomised-control trials	Cochrane Risk of Bias Tool
Non-RCT intervention studies	CASP Cohort Studies or Case-Control Studies Checklist or Cochrane risk of bias tool if an appropriate version is available
Qualitative studies	CASP Qualitative Study Appraisal Tool
Grey Literature	ACCODS or if a study the appropriate tool for the methodology was used.
Economic evaluations	CASP Economic Evaluations Checklist

#### Data abstraction and synthesis

We used the following methods and processes for the within stream and overarching syntheses of evidence:

- Cochrane Effective Practice and Organisation of Care Group [15] guidance for the synthesis of effect evidence;
- Cochrane Qualitative and Implementation Methods Group [16] guidance for the synthesis of qualitative and descriptive process evaluation evidence.
- Campbell and Cochrane Economics Methods Group guidance for the synthesis of cost and cost-effectiveness evidence [17].
- The framework described by Oliver et al [18] was adapted to juxtapose evidence in an overarching synthesis to determine whether targeted interventions mapped onto the expressed needs and problems experienced by young people.

The initial literature scoping suggested that there were insufficient randomised controlled trials (RCT) to undertake a meta-analysis of interventions. We therefore reported using a narrative summary approach. Where non-RCT quantitative studies had been undertaken, tables were developed to summarise baseline data, post-intervention results for both study and control groups along with a summary of risk bias and provide a narrative summary. For qualitative studies we conducted a thematic synthesis informed by the framework approach used for the analysis of primary qualitative data and adapted for evidence synthesis [19]. An *a priori* thematic framework was developed to identify index codes relating to the transition between paediatric and adult health care for young people with kidney disease using Bronfenbrenner's (1979) socio-ecological conceptual model as a starting point. The identification of such key constructs enabled the rapid coding of study data [20]. The preliminary framework was discussed within the research team. The thematic framework was further adapted using the themes and ideas that emerged through the synthesis. The emerging themes and concepts were collated in analysis tables, so that the columns and rows of the table reflect the studies and sub-themes [21]. The tables (e.g. Table 3) enabled the team to compare the results obtained in different studies across different themes and sub-themes and to compare the findings of different studies for each theme. Following the initial synthesis, a table of evidence (Table 6) brought together all the evidence and an overarching narrative synthesis was conducted across the streams of evidence, juxtaposing the issues and problems faced by young people with kidney disease and the interventions that may, or may not enable them to successfully transition between child and adult services.

**Table 3:** Stream one summary. The specific transition needs and problems experienced by young people with CKD.

Phenomena of interest	Propositions	References
**Social**	Adolescents and young people with CKD face difficulties in developing social networks.	[33,35]
	Side-effects of medication causes distress amongst adolescents and young people.	[33]
	Young people with CKD have a lower quality of life both-health related and generally.	[28]
	Young people with CKD are invisibly disabled.	[33]
	Psycho-social factors affect compliance.	[8]
**Developmental**	The age of initial diagnosis and transplant has a significant effect on transition needs	[23,57]
	Young people with CKD may suffer educational and subsequently employment disadvantages	[31,33–35]
	Restricted growth can decrease HRQOL for young people with CKD	[51]
	Young people with CKD show delays in achieving adult independence	[22,34,58]
**Psychological**	Young people with CKD may have cognition deficits.	[35]
	Young people with CKD suffer from low self-esteem.	[35,58]
	Depression and mental illness comorbid with CKD in adolescence.	[23,31,35]
**Health related**	Young renal transplant patients have higher risk of graft loss	[8,26,27]
	Adolescents and young adults with CKD at risk for poor health outcomes related to self-management.	[28] [58]
	Young people with CKD or kidney transplants have lower HRQoL scores	[37]

### Findings

We found that while intervention strategies to support transition contained consistent messages concerning the essential processes of transition, they generally support the principle of a health-led and dominated pathway. There is a lack of research that takes a holistic view of specific transition needs and issues for young people with CKD. Well-being is mainly defined and measured from a clinical perspective and the transition process is often set out as a linear, one-dimensional conduit with individual characteristics, along with social, familial and societal relationships rarely being considered. We discovered few interventions to support young people with CKD through transition and even fewer that had been evaluated, or that appeared to have been developed with young people with CKD. We found no cost data for the identified interventions. There was an absence of studies conducted by allied health and social care professionals (e.g. renal social workers and psychologists) who are integrated into the multi-disciplinary renal team. There was little cross-disciplinary research activity. Findings from each stream of evidence are reported in the following sections.

### Stream one: The specific transition needs and problems experienced by young people with CKD

Of the studies included within this stream of evidence (Table 3), six dealt with medical problems associated with graft loss from the perspective of clinicians [22–27] and suggested an age-related high risk of graft loss during the transition period. Primary reasons were increased vulnerability of adolescents and young adults, and a lack of medical adherence. Two studies [24,27] emphasised specific problems of the transfer from child to adult care with adherence improved when a single transfer clinic was available. Five studies [2,28–31] approaching the subject from both medical and social care perspectives highlighted educational issues for self-management and treatment adherence. Studies discussed the need for continuing information, explanation, and education regarding the treatment and the importance of adherence. Murray et al. [31] pointed out negative effects of dialysis treatment on academic education and work, finding that due to their treatment, patients miss time and struggle with a lack of understanding from educators and employers.

Six studies [8,32–36] addressed social and condition-related issues. Social aspects included difficulties in forming social networks related to a desire to appear ‘normal’; an overall lower quality of life that is also related to side effects; a delay in achieving adult independence; and restrictions on social and physical activities and day-to-day-life. Condition-related factors included the necessity of continuing treatment and follow up of CKD from childhood on to avoid a risk of relapse, the negative impact of comorbidities on the quality of life, and the need for effective coping strategies for young people.

Several studies [8,23,28,29,31,35,37,38] investigated psychological, developmental, health-related, institutional, socioecological, and vocational issues. Psychological issues for young people with CKD included cognitive deficits, low self-esteem, loss of independence, and loneliness. The age of initial diagnosis and transplant, educational and employment disadvantages, and restricted growth are all reported as having an impact on the development and quality of life. According to two studies [28,37] health is affected by an overall lower health-related quality of life, even if young people are higher educated and integrated in social life. There is also a risk of poor health outcomes due to deficiencies and barriers to self-management [28,29]. Institutional issues referred to the insufficient implementation of consensus guidelines regarding the transition process in children’s units. One study [38] also highlighted socioecological factors such as age, insurance status or ethnic origin as indicators for greater healthcare utilisation. Vocational issues, that often seem to be closely linked to educational or developmental issues, demonstrate that unemployment or low paid work is common in adults who survived childhood dialysis or transplantation. In addition, one study [31] reported a negative effect of dialysis on career ambitions and the effect of the treatment on young adults’ attendance at work.

### Stream two: Effectiveness and wider impacts of interventions to support transition for young people with CKD

We found no trials or economic evaluations and were unable to determine the effectiveness, or cost effectiveness of the eight transition intervention evaluations examined. Included studies (Table 4) were generally non-randomised local service evaluations with low participant numbers. Outcome measures used were often restricted to patient satisfaction [39], short-term clinical measurements such as clinic attendance [28] or graft rejection during the transition period [6]. Young people commonly declined to take part in evaluations. Of the more comprehensive interventions, there were two broad approaches set out. Firstly, bespoke ‘Joint transition clinics’ [6] or ‘transfer clinics’ [27] that bridged child and adult services were found to have short-term outcomes in terms of adherence and renal function yet studies did not measure any non-clinical outcomes for young people. Two studies [40,41] evaluated process-driven interventions that included defined ‘pathways’ designed to support young people through the transition process. Yet in these approaches there is very limited evidence of long-term impact either clinically, in quality of life or in the successful situatedness of young people in the adult world.

**Table 4:** Stream two summary: Effectiveness and wider impacts of interventions to support transition for young people with CKD.

Intervention	Reference
Young adult renal units	[6,59]
Multidisciplinary renal team	[6]
Starting transition in early adolescence	[40]
Transition policy	No evidence
Individualised transition plans	[41]
Assessments of transition readiness	No evidence
Better information for young people	[41]
Importance of young people meeting adult team before transfer	No evidence
Promotion of autonomy	[60]
Health worker education	[39,41,61]
Joint clinics with staff from both children's and adult services	[6,41,62]
Transition youth worker	[6,41]
Communication between children’s and adult services	[6]
Youth-centred planning	No evidence
Peer support	[6,41,44]
Residential camps	[6,63]

Several interventions [41–44] focused on peer-mentoring as a key component in supporting transition. Whilst young people with CKD often claimed that a peer-mentor (someone who has been through the same issues they have) would be beneficial when transitioning, studies reported conflicting findings, with some suggesting that it is not always welcomed by young people wanting to be ‘normal’ (e.g., [43]).

One study reporting application of the 'Readiness to transition questionnaire' [39] concluded that the young people believed they were ready for transition at an earlier age than their health care providers and parents did. This highlights one of the key conflict areas within the transition process with the age at which young people begin to transition is often an important period for young people in general, where they seek to break away from their parents and gain some form of independence, while parents, who have hitherto been their advocates and guardians, struggle to let go [39].

### Stream three: The views and experiences of young people with CKD and their parents/families and professionals of transition generally, and interventions to support transition specifically

Of the eight included studies only four were peer reviewed journal articles, three were grey-literature reports and one, a Thesis. Most contained a significant element of qualitative research and smaller cross-sectional survey data. Across all eight studies, the total number of participants was 147.

Findings (Table 5) charted the interactions between the young person, the Micro, Exo and Macro-systems [12] and the conditions of transition thereby identifying from the perspective of those experiencing transition, the elements that can hinder or support successful transition [45].

**Table 5:** Stream three: The views and experiences of young people, parents and healthcare professionals mapped against two-dimensional theoretical framework.

Environment	Transition conditions										
	Empowerment	Information	Communication	Independent support	Mutual / peer support networks	Education support	Handover between child and adult services	Transition Timing	The role of parents	Adult clinic environments	Independence and adherence
**Individual (Young Person)**	Few patients felt prepared for transition [46]	YP were 'told the truth' more readily in adult clinics which they found 'scary' but appreciated openness which for some was overdue [49]	Channels need to be engaging and tailored to YPs’ needs [48]	Experienced support workers valued by YP as friends dispensing non-clinical, non-family advice [52]	Less formal support networks—around understanding and sharing experiences—valued by YP [48] [52]	The age of onset, the timing of disruption, and the cumulative effect of long and frequent disruptive spells, predict educational outcomes. Children suffering from CKD pre-puberty have poorer attainment [33,47]	YP are concerned about not knowing what to expect in adult clinics, or have misconceptions [49]	Timing of transfer should be an individual choice based on maturity and 'being ready' [49]	YP want their parents to be actively involved during transition [46]	Few YP rated adult clinics highly [46]. Lack of pastoral assistance in adult care [52]. Impersonal and brutal [47] "Felt like being dumped" [50]	Non-compliance mainly attributed to 'forgetfulness' but alcohol and peer pressure also noted [49]
	YP with CKD have to rely on parental support more than their peers [52]	Info for YP needs to be tailored to life-changes outside of their health needs such as coping at college / university. Lifestyle info. becomes increasingly important with maturation [50]	Centring on the care of the YP means tapping into their most influential sources, such as friends and family. [52]	Non-clinical support workers valued highly by YP as providers of support and pragmatic advice. Holistic support—other areas of life—non-health issues [52]	Access to other young renal patients is essential [52]	YP with CKD diagnosis before age 12 suffer from poor educational and employment outcomes [33]	72% of YP with CKD did not meet anyone from adult service before transfer. 24% said they would like to meet new hospital clinicians [50]	YP talked about being 'thrown out' of paediatric settings [48]	Most YP appreciated taking more of a role but with reassurance of parental 'back-up'. [49]	YP feel that clinics don't realise that they have a life outside the illness. Jobs, social lives etc. can be seen as more important than appointments [48]	Increasing exertion of agency leading to risk taking for some YP, others more cautious. [47]
	Struggle between YP taking more responsibility and parents wanting to retain control is the key battle [52]		Under 18's rely more on websites and Facebook. They don't respond to information from HCP's. Older YP prefer to receive info from HCP's [50]		35% of YP would like to meet other YP with CKD. 32% would not (Woodland 2015)					For the YP, a new adult hospital where they are now the youngest patient and are sitting in the waiting room with older kidney patients is very daunting; it feels different, familiar people are not present to comfort them, and new processes aren’t explained in the way they are used to. [48]	Adult clinicians perceived to be taking punitive approach to non-adherence [47]
	Some YP feel powerless about being told at what age they can manage their condition, while others worried about taking responsibility for their conditions with life-threatening consequences [47]	Learning from other patients of a similar age about CKD and treatment [51]			Participants felt more confident socialising with peers who had CKD. Some felt that hearing other patients' stories enabled them to positively reflect. [2]					YP may feel less important at local adult unit. Turnover of HCP's and lack of familiarity can be alarming. [53]	
	Depression and lack of emotional support likely to delay progress towards autonomy [47]				Being with peers with similar experiences alleviated emotional burden [51]					45% of YP over 18 would like to attend a young adult clinic. 28% would not. [50]	
							YP expressed significant anxiety about appointments, blood tests and biopsies in adult clinics [49]			YP put off by adult clinics being 'full of old people' [49]. Hopes of a normal life ahead dashed by seeing older, sick renal patients [47]	
**Microsystem (Family, peers, health practitioners)**	Children’s services balancing autonomy with parental involvement during transition [46]	Children’s services did not always equip YP with the knowledge and skills required to negotiate health systems [46]	YP needed time to talk about transfer with doctors and nurses [46]	Experienced renal support workers can provide families with important emotional support during transition. [48]	Parents often feel isolated after transition. Support networks of other CKD experienced parents needed [48]		YP need to 'feel handed-over' into care of adult team [49]	Decisions about timing of handover imposed by health professionals. [46]	Parents perceive practices in adult clinics as lack of continuity of care and become concerned [48]	Staff less personal, sometimes less sympathetic in adult clinics [49] Abruptness [52]	HCP's and parent's voices less likely to be heard during adolescence [53]
	YP emphasised the importance of a gradual shift in responsibility [49]	Parents say there is little to no guidance on helping a child with a long-term condition during transition [48]	Doctors in adult clinics expected them to have more knowledge about their condition [49]	Some YP dismissive or skeptical about pyschological support / counselling (mental illness stigma?) [47]	Renal support networks aren't valued as highly as friendhip groups (online or otherwise) [52]		Moving into adult care often means loss of broader family support package [48]		Siblings suffer. Lack of attention can have very negative consequences on family life [48]	Families of CKD YP feel excluded even though they have wealth of knowledge and experience [48]	Conflicts between peer acceptance and treatment / medication regimes. Being labelled 'sick' set them apart. [64]
	Discreet parental support important but parents regaining control after set-backs can be dis-empowering. [48]		As YP get older they increasingly rely on friends for support, advice and information, but friends are not well-informed. Reliance on internet for information can be problematic [52]		Family support gradually replaced by support from friendship groups and / or partners [47]		Initiating a partnership between the parent and hospital will be important in ensuring the young adult has rounded support, and for the parent it is reassuring to have confirmation that they are carrying out their role [48]		Parents can struggle with the tension between pushing the young adult to be independent and being a protective parent [48]	Adult clinics focus on the autonomous individual and struggle to cope with strong parental involvement [53]	
					For some, providing peer support gave them a sense of fulfilment [51]						
	While YP appreciate being treated as an adult, they wanted some allowance made for their young age [49]				YP did not want support from other patients, especially older patients, or to make friends with their peers with ERF; they wanted 'normal' friends and eschewed those who were ill. [47]		Parents and YP would like a formal handover between child and adult services to include case history, roles and responsibilities [48]		YP positive about being able to talk more openly without parents but recognised that parents may find this change difficult [49]		
**Exosystem (family support networks, policy, funding)**	No matter what their age, parents find it difficult to step away [48]		Many YP transferred did not know the name or contact number of person at adult clinic on their first visit [46].				Co-ordination between hospitals and importance to YP about 'feeling handed-over' [49]	Instances of inflexible policy of transferring YP at 18 [46]	Parents of YP with CKD feel their experiences and needs are unique and find ‘general support’ (counselling from the GP for instance) to be of little use, as it doesn’t take into consideration the multi-faceted and on-going struggle they feel they face [48]		
	YP may feel embarrassed to go to an adult clinic with their parents [53]	More information about different 'care culture' of adult clinics needed YP want their parents to be actively involved [46]						Children’s services need to accept that not all patients are ready to transfer at age 18 [49]		Culture of adult clinics de-humanising and lonely [46]	
**Macrosystem (Societal norms, culture, ideologies)**	Dependant on their individual charactersitics and comorbitities, YP with CKD may be more or less capable of fitting with societal norms around taking responsibility for themselves. [49] [48] [46]							The societal expectation that at age 18 children become adults does not necessarily sit comfortably with the needs of this group of YP [49] [48] [46]			External pressures—exams, social life—impact on adherance [49] [53]

#### A. Empowerment

Empowerment was reported as being the key transition battleground with protagonists being the young person, their parents, health care practitioners and health systems. Studies [46–48] concur that few young people feel prepared for transition. Depending on their individual characteristics and comorbidities, young people with CKD may be less capable of fitting with societal norms around taking responsibility for themselves. Some young people feel powerless about being told when they can manage their condition, while others worry about taking responsibility for their conditions with life-threatening consequences [47].

#### B. Information

Young people with CKD said that they needed a range of information to support their transition but few seemed to receive the information that they required to navigate clinical and broader life changes, with an acceptance that these are co-dependent [49–51]. Children’s services rely on parents and do not always equip young people with the knowledge and skills required to negotiate health systems [46]. Changes to young people’s lives such as leaving home, going to university or starting a job can and will, have an impact on young people’s health management [50]. Parents also said that there was little information for them providing guidance on helping a child with a long-term condition during transition. Once a young person was transferred, the different ‘care culture’ of the adult clinic resulted in parents being shut out despite young people often wanting their parents to be actively involved [52]. Despite the change in culture, young people appreciated the directness of the information being given by adult clinics but could also be ‘scared’ by bare truths related to their condition [49].

#### C. Communication

The quality of communication between different health settings and patients was reported as highly variable during transition. At the same time, with increasing age young people relied more on informal sources of communication away from HCP’s and family, and towards friends and online communities with consequent concerns around the quality and accuracy of messages [50,52]. Young people said that they needed time to talk about transfer with HCP’s but once they transferred to adult clinics around the age of 16–18 years, they were expected to have more knowledge about their condition than they did [46].

#### D. Independent support

A common feature of the interventions identified in Stream Three was the introduction of a transition or non-clinical peer support worker to help young people through the process [48,52]. Young people were generally very positive in the evaluations of these interventions. Being non-clinical, support workers were better able to take a holistic view of the change from childhood to adulthood accepting that life-changes were inextricably linked with health changes. Nonetheless, others were reported as being dismissive or sceptical about psychological support or counselling with stigma around mental illness being a possible cause [47].

#### E. Peer support

The efficacy of wider peer support networks is unclear. In one survey [50] equal proportions of young people said that they would like to meet other young people with CKD as would not. While in qualitative studies [48,51] young people said they valued support networks as a way of understanding and sharing experiences, in others they did not want support from other patients, especially older patients, or to make friends with their peers with CKD they wanted 'normal' friends and eschewed those who were ill.

#### F. Education

One study [33] examined the impact on educational and employment outcomes of young people with CKD finding that the age of onset, the timing of disruption, and the cumulative effect of long and frequent disruptive spells, predicted educational outcomes, with children suffering from CKD pre-puberty having poorer attainment. In other studies [36,53], the pressure of school work and exams was cited by young people as an issue in adherence, again highlighting the need to take into account health transition within the wider context of change in young people’s lives.

#### G. Handover between child and adult services

Some young people experienced a limited hand-over between child and adult services. Young people expressed significant anxiety as a result [49,50]. They consistently said that they wanted to be formally handed-over in a process where they received holistic transition support over a longer period of time. Parents also wanted to be reassured that they could cope as moving into adult care often meant loss of the broader family support package.

#### H. Transition timing

Young people talked about being 'thrown out' of children’s settings once they reached 18 years of age with some highly inflexible practices [49,54]. Young people felt that the timing of transfer should be an individual choice based on maturity and 'being ready', thereby suggesting that for this group, the societal expectation that at age 18 children become adults does not necessarily sit comfortably with their needs.

#### I. Parental roles

One study [52] that focused on the role of parents found that they can struggle with the tension between pushing the young person to be independent and being a protective parent. Young people wanted their parents to be actively involved during transition but appreciated taking more of a role but with reassurance of parental 'back-up'. Within the adult clinic young people said they found it positive to be able to talk more openly to HCP’s without parents but recognised that parents may find this change difficult. Parents who up until this point had tended to be fully in control of their children’s healthcare needs in the supportive children’s environment struggled to cope with the abrupt change brought about in transferring to adult clinics.

#### J. Adult clinics

Few young people rated adult clinics highly [46–48,55]. They criticised the lack of pastoral assistance in adult care, some calling them impersonal and brutal and one saying that transition "…felt like being dumped" [52]. Hopes of a normal life ahead were also affected by seeing older, sick renal patients and young people said that health staff were less personal and less sympathetic, with little allowance made for their age (ibid).

Families of young people with CKD said that they feel excluded in adult clinics even though they have wealth of knowledge and experience while adult clinics focus on the autonomous individual and struggle to cope with strong parental involvement [52]

#### K. Independence and adherence

Some young people attributed non-compliance to forgetfulness brought on by the external pressures of a developing social life, changing life-priorities or alcohol use [49] For others, non-compliance was the exertion of agency leading to risk-taking [47]. Young people were often conflicted between peer-acceptance where ‘being sick’ sets them apart and the medication and regime messages from health professionals and parents that are less likely to be heard as young people mature. Where young people did not comply with treatment, some reported that adult clinics were more likely to take punitive measures against them [47]

### Overarching synthesis

Conducting an overarching synthesis using Oliver et al.’s [18] framework (see Table 6) to juxtapose evidence on similar phenomena within a theory-driven, two-dimensional lens enabled us to understand that transition conditions alone are unlikely to facilitate success. It is the interactions between the person in transition and the conditions they experience that determines success [45]. Evidence suggests that a ‘successful’ transition is conceptualised differently by clinicians, young people and their families. There was a good level of consistency regarding many ‘conditions of transition’ [45], but while these are identified as factors that facilitate or hinder transition in the literature, they focus only on health system approaches. While many of the studies report the different experiences of transition experienced by young people—and go some way to recognise the importance of flexibility—the principle of a linear health-dominated pathway remains. This is clearly misaligned with the needs expressed by young people.

**Table 6:** Integration of evidence across the streams [18].

Stream 1: The specific transition needs and problems experienced by young people with CKD	Stream 2: Effectiveness and wider impacts of interventions to support transition for young people with CKD	Stream 3. The views and experiences of young people with CKD and their parents/families and professionals of transition generally, and interventions to support transition specifically	Gaps
Psychological, developmental, health-related, institutional, socioecological, and vocational issues.	Absence of evidence of the effectiveness or ‘added value’ of integrated health and social care renal teams.	Young people want holistic transition support over a longer period of time.	There is an absence of evidence on the role and impact of integrated health and social care renal teams. The evidence is medically lead and interpreted through a medical lens. There are virtually no studies published from the perspective of renal social work and social care.
Information, explanation, and education regarding the treatment and the importance of adherence.	Very limited evaluation of interventions and no evidenced consensus regarding timings.	Depending on their individual characteristics and comorbidities, young people with CKD may be less capable of fitting with societal norms around taking responsibility for themselves.	Transition is not consistently conceptualised across disciplines. There is an absence of evidence that starting transition early improves outcomes
Importance of social factors during transition.	Pathway interventions evaluated have been process-driven with few non-clinical or long-term evaluations.	Transition should be individualised. Some feel powerless about being told when they can manage their condition, while others worry about taking responsibility for their conditions with life-threatening consequences.	Key conflict / resolution area with little evidence that existing interventions are meeting the personalised clinical *and* social-care needs of young people, and long-term outcomes remain unknown.
Attachment of young people to children’s units.	Found to have short-term outcomes in terms of adherence and renal function but no measures of non-clinical outcomes for young people.	Children’s services rely on parents and do not always equip young people with the knowledge and skills required to negotiate health systems. Adult clinics viewed very negatively. Families excluded.	Some evidence of positive outcomes but further long-term evaluations of different interventions needed to develop good practice.
Risk of disengagement, isolation, and subsequently poor health, social, educational and vocational outcomes.	A number of small-scale evaluations using a variety of approaches but limited assessment of outcomes.	A broad range of information support wanted by young people that goes beyond clinical and health needs. Pressure of school work and exams cited by young people as an issue in adherence.	Limited evidence of long-term impact from programmes either clinically or socially. The health and social care needs of young people with CKD in transition are poorly understood.
YP suffer cognitive deficits, low self-esteem, loss of independence, and loneliness.	Peer support and non-clinical mentoring trialled with some evaluation.	Peer-support and mentoring appreciated by some young people, rejected by others.	Paucity of studies and small cohorts makes evaluation difficult and therefore developing best-practice problematic.

We found that the well-being of young people with CKD was most often defined and measured in clinical terms. This would seem to provide little opportunity for the uniqueness of individual to be considered. Many of the studies dealt with medical issues focusing on the outcome of age-related graft adherence with an emphasis on service delivery reform as the key to improving graft loss and tackling non-compliance complications. They often examined the narrow clinical environment within which the young person interacts without considering the characteristics of the young person nor their wider social care needs. Biological factors, functional limitations and awareness of the transition are all important in the young person’s ability to develop self-awareness and self-management skills [45] that allow them to move towards mastery of their condition and therefore need to be considered as facilitators or inhibitors of transition. Yet at the same time, they are equally important in the broader developmental journey into adulthood that is set within social, family and societal relationships. Without consideration of what Bronfenbrenner [11] calls the *proximal processes* where the interactions between the person and their environment are effectively aligned, successful transition-related goals are unlikely to be realised.

Socially, it seems that young people with CKD face greater difficulties in developing networks and can face barriers to participation in day-to-day social activities at a time when this is becoming central in their lives. Studies have identified that treatment regimes, side-effects and comorbidities all have a part to play in non-compliance but few set these issues within the wider developmental context of the young person’s agency. Similarly, young people with CKD face additional psychological challenges. Poor self-esteem, loneliness, depression and cognitive deficits are commonly reported as resulting in poor quality of life, increased vulnerability and both negative health (including graft-loss) and social outcomes. There is also some evidence of longer-term effects on adult life chances through poorer educational and employment opportunities. Nonetheless, what is missing from existing evidence is a better understanding of how young people’s neurobiological, physical and developmental trajectories intersect with the contextual changes provided through the transition from child to adult care.

Evaluations of the interventions studied suffered from small samples and in some cases, low participation rates of young people which may be explained by evidence from other sources [39,44] that young people with CKD are often in denial about their illness. While these young people have specific vulnerabilities (such as losing their transplanted kidney due to inappropriate self-management), they are difficult to engage with consistently. Our overarching synthesis exposes gaps that exist between the social care needs of young people within the multiple environments they inhabit and the transition conditions identified in the evaluation of literature. It becomes clear that transition is a conflict zone. The balance between independence and dependency is being played-out on many levels, from young people’s own internalised struggles between feeling powerless on one hand and overwhelmed on the other, to the need for support yet the requirement to make autonomous decisions, to facing up to living with what can be a life-limiting condition alongside the expectations of wider society and their peer-group.

The design of interventions to date has not brought together consideration of these conflicts within developmental and service delivery transitions or followed the Medical Research Council guidance for the design and evaluation of complex interventions. As Joly [45] sets out, effective interventions that integrate transition and bioecological needs require an ambitious overarching outcome that incorporates medical stability, the best quality of life possible, along with mastery or new skills and behaviours needed within new environments and movement towards life goals. The overarching synthesis suggests that a new approach is needed to designing young-person centred interventions to support their identified transition needs.

## Discussion

Findings confirm that young people with CKD share some common transition issues with other long-term conditions such as diabetes [56]. Common issues include medication and treatment adherence, disengagement from services and risk-taking behaviour that jeopardises their kidney health and overall wellbeing. Although some factors that contribute to poor outcomes appear to be beyond the control of young people (such as their immune system), other issues such as optimal self-management to preserve their remaining kidney function or to protect their transplanted kidney are within their control. The stark consequence in CKD is that in taking risks and disengaging from optimal self-management young people are commonly putting their kidney health at risk—expediting the need for a kidney transplant—or they are risking the loss of their transplanted kidney, both of which have severe consequences for their treatment options, life experience and their life expectancy. The avoidable loss of a transplanted kidney also raises some ethical and moral issues about lack of respect for the deceased donor and their family.

To date, we find that studies have primarily focused on condition-related, treatment related or socio-economic determinants for non-adherence amongst adolescents and young adults with CKD (we include a number of such studies as relevant, but which are not cited in the stream analyses—see S1 Table [65–88]) while patient-related and health-care system factors have not been well studied. Although there have been studies that propose or evaluate interventions to improve transitions for CKD patients, as a number of authors highlight [4,5,7,8] there is a significant gap in research that first, acknowledges that transition is a multi-layered concept involving patients, their families, health and social care providers and health and social care systems–all operating within a cultural, political and condition-related context—and secondly evidence of the effectiveness of policies, interventions and strategies aimed at supporting transition for young people with CKD. This results in a paucity of evidence at every level that is available to reliably inform providers of health and social care services about how best to meet the needs of this small but vulnerable cohort and provide them with the best life chances.

## Research prioritisation

Whilst research priorities should be fully developed with input from young people, families, health and social-care practitioners, based on the findings of this Review a broad programme of research is needed to fill gaps in current knowledge and inform service development and is set out in Table 7.

**Table 7:** Research priorities.

Knowledge gap	Perspective	Research activity	Outcomes
A clearer picture of the health and social care needs of YP with CKD as they progress from childhood through adolescence to young adulthood.	Psychological, health-related, vocational, developmental, institutional, and socioecological issues. Accounting for factors such as culture, ethnicity, economic circumstances, health and social care system, mental capacity, family capacity etc.	Qualitative research programme engaging YP with CKD, families, health and social care practitioners. Longer-term evaluation of health and social care outcomes of transitioning YP with CKD	Evidence to inform service development and practice across health and social care.
Effectiveness and outcomes from current health and social care practices including transition readiness and planning; multi-disciplinary working; service configurations; and support programmes.	Practitioner-led within disciplines across health and social care.	Qualitative evaluation and reflections and / or action research examining current practice.	Improvements within individual disciplines and identification of new opportunities for integrated working.
Effectiveness of trialled interventions for YP with CKD in integrated health and social care.	Multi-agency and integrated services including health, social care, education, employment, housing etc.	Longitudinal evaluation or long-term case studies from an integrated health and social care perspective involving YP and their families.	Identification of working practices most effective in transitions and movement towards individual life goals.
Development and trial of new evidence-based, novel transition programmes or components	The health and social care needs of YP with CKD during their transition to adulthood	Evidence-based intervention development Randomised controlled trials. Long term evaluations.	Improved medical and social practice and better outcomes for YP with CKD

### Strengths and limitations of the study

An important limitation of this study is that chronological age does not always match with maturation and developmental age. This is particularly important when children and young people with kidney disease also have developmental delay. Developmental age is the age at which the child or young person functions emotionally, physically, cognitively and socially [89]. The challenge in a systematic review context is that the reviewer is usually only provided with age and gender and is not able to determine if participants also had developmental delay. Therefore when applying the findings, practitioners should be aware of the need to be flexible in their use of age-appropriate evidence and ensure that they make appropriate adaptations for their clients whose developmental age is different to their chronological age.

Although our search was rigorous and we used a purposive sampling strategy, systematic searching of all sources on all potential terms was not possible within the time frame. However, a wide range of databases and further seed pearling was used to achieve maximum variation of potential sources of evidence. The paucity of published research in this area meant that qualitative studies were more likely to be excluded because of their lack of relevance to the research questions than on quality grounds. Study quality assessments were considered within the synthesis and any issues that may impact on findings noted. The lack of controlled trials and low participant numbers has consequences for risk of bias and confidence in findings. One of the strengths of this review is in identifying such gaps for further investigation.

## Conclusions

In deploying social theory this review provides an interpretation of available evidence that portrays some of the complexities that practitioners must account for in planning successful transitions for a vulnerable group of young people. In particular, the holistic needs of young people with CKD need to be recognised including condition-related, social and psychological factors, alongside maturation. Furthermore, as theory [11] predicts, the concept of ‘success’ in transition, while being assigned objective properties by clinicians and others, is often defined subjectively by young people themselves, contextualised by the ecosystems they occupy. These multiple environments need to be understood and accounted for if transitions are to meet young people’s needs, yet current practice appears to be misaligned, dominated by linear health-related pathways.

We conclude that structurally, the gap in culture and practice between child and adult services is often too big for young people to negotiate. Integrated health and social care renal services need redesigning to better accommodate the influence of individual and socio-ecological factors. We need to better understand why young people risk their kidney health or kidney transplant in order to develop transition services and interventions using evidence-based principles and with the input of young people themselves. Effective mechanisms for engaging young people in research and service development need to be established. Future research needs to broaden beyond a medical lens and be led from alternative multi-disciplinary and social care perspectives, and by young people themselves.

## Supporting information

S1 Table Summary of included studies. (PDF)
