# Supplementary material for: Why is transition between child and adult services a dangerous time for young people with chronic kidney disease? A mixed-method systematic review
Source: PLoS One. 2018 Aug 2;13(8):e0201098. doi: 10.1371/journal.pone.0201098 (PMC6071995; doi:10.1371/journal.pone.0201098)
Supplement: S1 Table — (PDF) [file pone.0201098.s001.pdf]

S1 Table: Summary of included studies

Author and Reference	Context	Study Design	Country	Synthesis stream	Focus / Research question	Sample
**Aasebø et al. (2009)** [37]	Patient outcomes	Cross-sectional survey	Norway	S1	Quality of life of young transplant recipients	131 Young adult (18–35 years of age) transplant recipients
**Akchurin et al. (2014)** [65]	Clinical study	Cohort Study	US	S1	Whether adherence to immunosuppressant medications changes during transition from a pediatric to an adult program within the same transplant center	24 patients studied in one transplant centre
**Andreoni et al. (2013)** [25]	Clinical study	Cohort Study	US	S1	US study of graft loss	
**Bell H, Hope W (2012)** [66]	Clinical intervention	Mixed methods	UK	S2	Improve clinical outcomes for young adults (aged 18-25) with CKD	Unclear
**Chaturvedi, Jones, Walker & Sawyer (2009)** [46]	Research Protocol	Cohort Study	UK	S3	Surveying of people experiencing young adult kidney failure	UK young adults aged 16-30 on chronic RRT in 2015 (n≈1000)
**De Ferris and Diaz-Gonzalez de Ferris (2011)** [1].	Clinical practice	Secondary data analysis	EU, North America, Australia, and New Zealand	S1	Unique Morbidities and Adherence Issues of adolescents and YP with CKD	15- to 24-year-old patients, 7,000 adolescent/emerging-adult dialysis patients and 11,000 renal transplant recipients
**Ritchie, Clayton, Mackie et al. (2012)** [67]	Patient outcomes	Cross-sectional survey	Australia	S3	Satisfaction survey combined with clinical records of success	11 patients
**Department for Health (2006)** [68]	Policy statement	n/a	UK	S2	National Service Framework for renal services: working for children and YP	n/a
**East Midlands Renal Network (2012)** [41]	Intervention review	Mixed methods	UK	S1, S2	young adults aged 18 – 25 years with chronic kidney disease	80 young adults in baseline, 76 after 12 months
**Ferris et al (2015)** [28]	Intervention proposals	n/a	US	S1, S2	Adolescents/young adults (AYA) with chronic kidney disease (CKD) or end-stage kidney disease (ESKD)	n/a
**Forbes et al. (2014)** [29]	Clinical intervention	Cross sectional survey	Europe	S1	Transition and transfer of young adult patients from paediatric to adult renal units	15 centres
**Foster et al. (2011)** [57]	Clinical study	Cohort Study	Canada	S1	Research looking at impact of age at transfer on renal allograft failure rates	440 kidney recipients who had been transferred from pediatric to adult care
**Gilleland Marchak et al. (2015)** [39]	Clinical intervention	Mixed methods	US	S2	Evaluation of Readiness for Transition Questionnaire (RTQ) for transition readi-ness perceptions, adolescent healthcare behavior, and familial involvement in health care for CKD patients	49 kidney transplant patients
**Guy's and St Thomas' NHS Foundation Trust (2012)**	Patient's self-management	n/a	UK	S2	Support of self-management and kidney care record	n/a
**Hamilton et al. (2015)** [69]	Patient satisfaction	Focus group	UK	S3	Aspects of the transition highlighted by YP. Compliance as measure.	Ten participants aged 18 to 23 years with CKD and three parents.

**Hannigan & Turner (2011)** [49]	Opinion study	Ethnography / Case Studies	UK	S3	The views of parents of young adults with CKD	Four interviews with parents
**Harden et al (2012)** [6]	Quality improvement report	n/a	UK	S2	Report on effectiveness of young adult clinics	9 patients (direct transfer) and 12 patients (young adult services)
**Harden and Nadine (2006)** [55]	Case study	Case study	USA	S3	Draws on the personal experiences of patients who have made the transition from pediatric care to adult care	1 case study
**Harden et al. (2012)** [6]	Intervention evaluation	Cohort study	UK	S2	Before and after study of the impact of a new integrated paediatric-adult clinical service for patients with kidney failure	12 paediatric transplant recipients (seven male, five female) aged 17.5 (16–18) years
**Harden et al. (2010)** [70]	Intervention evaluation	Cohort Study	UK	S2	Conference report on the Oxford Young Adult Transplant Clinic	19 patients attending the Young Adult Transplant Clinic
**Iitaka et al. (2008)** [30]	Clinical Study	Retrospective Cohort Study	Japan	S1	Transition of patients with membranoproliferative glomerulonephritis (MPGN)	27 children all aged 18 at end of study
**Ipsos Mori / NHS Kidney Care (2012)** [48]	Clinical study	Qualitative study	UK	S3	To examine ways in which families cope with oral medication post-transplant	17 transplant patients and their caregivers
**Ipsos MORI (2013)** [52]	Opinion study	Ethnography / Case Studies	UK	S3	The views of YP with CKD	Six YP aged 20 to 26
**Javalkar et al. (2014)** [38]	Patient study	Cohort study	US	S1	Testing of TRxANSITION scale for readiness for self-management and transition.	52 adolescents and young adults aged 13 to 21
**Jorge et al. (2007)** [71]	Clinical study	Retrospective cohort study	US	S2	Evaluation of paediatric to adult ou-patient clinic	69 patients. Mean age 17.6 years
**Kiberd, Acott and Kiberd (2011)** [26]	Clinical study	Single center program retrospective chart review	Canada	S1	Evaluation of transfer process from paediatric to adult care.	Young adults 18 to 25 attending a single centre.
**Kreuzer et al. (2014)** [72]	Clinical Trial	Evaluation of Transition programme	Germany	S2	Evaluation of the current patient transition situation in all existing German pediatric and nephrology departments	17 centres
**Cameron et al. (2011)** [73]	Review	Review of transition issues	US	S1	Barriers to transitioning that	n/a
**Lewis and Arber (2015a)** [33]	Patient experience	Qualitative study	UK	S1	Gender, peer and intimate relationships amongst YP with CKD	Qualitative interview data from 40 young adults aged 16–30 years with end-stage kidney disease
**Lewis and Arber, (2015b)** [32]	Patient experience	Qualitative study	UK	S1	Link between age, renal failure and education and employment	35 qualitative interviews with end-stage renal failure patients, aged 20–30 years, first diagnosed at 0–19 years of age
**Lewis and Marks, (2014)** [34]	Patient outcomes	Cross sectional survey	UK	S1	Age of CKD presentation and effects of education and employment	Young adults (n = 296, 52 % male, 79 % Caucasian and 73% with functioning renal allograft) with a mean age at first presentation of ESKD and current age of 17 and 25 years,

**Lewis (2012)** [47]	Thesis study	Mixed methods	UK	S1, S3	Experiences of biographical disruption at critical moments in the lives of young adults with childhood renal failure.	40 patient interviews and 10 parent/guardian interviews
**McDonagh and Kelly (2010)** [74]	Research and policy gaps	Review of transition issues	US	S1	Challenges and opportunities for transitional care research	n/a
**McNulty (2014)** [75]	Intervention description	Conference presentation	UK	S2	Description of the Salford Adult / Transition Renal Clinic.	n/a
**McQuillan et al. (2015)** [27]	Retrospective study	Cohort study	Canada	S1, S2	Whether a transfer clinic for YP with kidney transplants improves adherence post transfer	16 kidney transplant recipients who transferred before the clinic was established and 16 from afterwards
**Mekahli et al. (2014)** [35]	Patient quality of life	Cohort Study	Holland	S1, S3	Quality of life of YP diagnosed with CKD in infancy	41 patients <16 years
**Murray et al., (2014)** [31]	Patient outcomes	Mixed methods	UK	S1	Impact of CKD on academic and employment outcomes.	57 young adults (ave. age 25)
**Myers (2002)** [76]	Intervention evaluation	Mixed methods	Australia	S3	Patient perspectives of a young adult renal clinic	15 patients
**Nagra et al. (2015)** [40]	Report on new transition programme	n/a	UK	S2	Discusses the benefits of a new programme to help those transitioning from pediatric to adult care.	n/a
**National Institute for Clinical Excellence (NICE) (2015)** [77]	Draft for consultation	n/a	UK	S2	A draft guideline for recommendations for transitions.	n/a
**Foster and Pai (2014)** [78]	Case study	Case study	UK	S3	Draws on the personal experiences of patients who have made the transition from pediatric care to adult care	2 case studies
**Lämmermühle, Oldhafer, Blume, Weiss, and Ahlenstiel (2013)** [79]	Retrospective study	Case control study	Germany	S1, S2	The use of a specialised transition clinic improved the level of patient satisfaction and aided the transition process.	66 patients. Data collected one year before and after transfer
**Remorino and Taylor (2006)** [80]	patient satisfaction	Qualitative study	UK	S2	Investigation of transitional clinics	18 patients
**Samuel and Nettel-Aguirre (2012)** [81]	clinical study	cohort study	USA	S1, S2	Adaption period to adult care associated with twofold risk in graft failure	413 patients with childhood-onset ESRD
**Samuel et al. (2014)** [82]	patient outcomes	cohort study	Canada	S1	Hospitalisation after transfer to adult care	349 patients divided in a cohort with transfer to adult care and 1 cohort solely cared for in adult care
**Sattoe et al. (2014).** [60]	Clinical intervention	Qualitative study	Netherlands	S2	Feasibility of intervention	participants of 'skills for growing up' tool
**Sattoe, Jedeloo, and van Staa (2013).** [63]	Peer-to-peer-support	Qualitative/mixed methods study	Netherlands	S2	Experience as buddy/attendee in the intervention	62 participants of 'Camp COOL'-programme
**Shaw et al.(2003).** [83]	Clinical study	Cohort Study	USA	S1	Risk of non-adherence, rejection, and graft loss for adolescents	112 transplant patient's at children's hospital
**Tong et al. (2015)** [51]	Patient outcomes	Mixed methods study	USA	S3	YP's experiences and perspectives regarding kidney disease, special focus on body image	10 patients from a large urban teaching hospital, purposive samples
**Van den Heuvel et al. (2010)** [22]	Clinical study	Cohort Study	Netherlands	S1	Correlation of age at transplantation and transition	cohort of 162 immigrant and Dutch patients

**Tong et al. (2011)** [84]	Patient outcomes	Mixed methods	Australia	S3	Experiences and perspectives of adolescent kidney transplant recipient	22 in-depth, face-to-face interviews with adolescent kidney transplant recipients (aged 12-19 yr) from five Australian pediatric transplant units
**Watson (2000)** [8]	Clinical study	Cohort Study	UK	S1	Transplant failure in young adults following transition.	20 young adults (9 female) who had been transferred to three different adult centres at a mean age of 17.9 years (range 15.7–20.9 years) having been transplanted at a mean age of 14.3 years
**Watson, Alan R (2011)** [85]	Patient information	n/a	UK	S1	Transition and transfer of young adult patients from paediatric to adult renal units	n/a
**Watson et al.(2011)** [86]	Consensus statement	n/a	UK, USA, Australia, Egypt	S1	Recommended action for transition process	n/a
**Wells, Ritchie, and McPherson (2013)** [58]	Patient Survey	Cross-sectional survey	UK - South West	S3	Views of YP and adolescents with CKD on transition.	Not clear. Two age groups - 14 to 18 and 18 to 25
**NHS England (2007)** [87]	Training materials	n/a	UK	S2	Renal Action Learning Sets	Records of Action Learning sets involving transition from teams at Birmingham and London.
**Woodland, Jo (2013)** [50]	Service Improvement	Qualitative study	UK	S3	Improvement of support for young adults	30 14-18 year olds (response rate 53%), 105 18-25 year olds (response rate 52%)
**Zheng, Kai et al (2010)** [88]	Patient satisfaction	Qualitative study	USA	S2	Feasibility of web community for ESRD patients	46 ESRD patients from 18 dialysis units
